# Content validation of an instrument for medical record audits

**DOI:** 10.1590/0034-7167-2022-0109

**Published:** 2023-10-09

**Authors:** Carla Simplicio, Ariane Polidoro Dini

**Affiliations:** I Universidade Estadual de Campinas. Campinas, São Paulo, Brazil

**Keywords:** Patient Safety, Medical Records, Validation Study, Total Quality Management, Clinical Audit, Seguridad del Paciente, Registros Médicos, Estudio de Validación, Gestión de la Calidad Total, Auditoría Clínica, Segurança do Paciente, Prontuários, Estudos de Validação, Gestão da Qualidade, Auditoria Clínica

## Abstract

**Objective:**

To build and validate the content of an instrument to conduct medical record audits; to conduct a pre-test.

**Methods:**

Methodological study conducted from May/2020 to May/2021 in three stages: 1) development of the instrument by bibliographic survey and benchmarking; 2) content validation using the Delphi technique; 3) application of the instrument and descriptive analysis in a sample of 200 medical records.

**Results:**

An instrument was constructed with 11 domains containing sub-items that characterize the quality of care. Two stages of the Delphi technique were necessary to reach a content validity index higher than 0.90. For each domain, a graduated scale with a numerical value from 1 to 4 points was attributed, reflecting the quality of its completion. The average time of application was 35 minutes per record.

**Conclusions:**

The tool proved to be viable to support clinical audits to identify the level of excellence and reveal opportunities for improvement in care processes.

## RESUMO

**Objetivo:**

Construir e validar conteúdo de um instrumento para realização de auditoria clínica de prontuários; realizar pré-teste.

**Métodos:**

Estudo metodológico, realizado de maio/2020 a maio/2021 em três etapas: 1) construção do instrumento por levantamento bibliográfico e *benchmarking*; 2) validação de conteúdo pela técnica Delphi; 3) aplicação do instrumento e análise descritiva em uma amostra de 200 prontuários.

**Resultados:**

Construiu-se um instrumento com 11 domínios contendo subitens que caracterizam a qualidade da assistência. Para chegar ao índice de validade de conteúdo superior a 0,90, foram necessárias duas etapas da técnica Delphi. Para cada domínio, atribuiu-se uma escala graduada com valor numérico de 1 a 4 pontos, refletindo a qualidade do preenchimento. O tempo médio de aplicação foi de 35 minutos por prontuário.

**Conclusões:**

O instrumento construído demonstrou-se viável para embasar a auditoria clínica na identificação do nível de excelência ou oportunidades de melhoria em processos assistenciais.

## RESUMEN

**Objetivo:**

Construir y validar contenido de un instrumento para realización de auditoría clínica de prontuarios; realizar pre-test.

**Métodos:**

Estudio metodológico, realizado de mayo/2020 a mayo/2021 en tres etapas: 1) construcción del instrumento por análisis bibliográfico y *benchmarking*; 2) validación de contenido por la técnica Delphi; 3) aplicación del instrumento y análisis descriptivo en una muestra de 200 prontuarios.

**Resultados:**

Construido un instrumento con 11 dominios conteniendo subitems que caracterizan la calidad de la asistencia. Para llegar al índice de validez de contenido superior a 0,90, fueron necesarias dos etapas de la técnica Delphi. Para cada dominio, atribuido una escala graduada con valor numérico de 1 a 4 puntos, reflejando la calidad del relleno. El tiempo mediano de aplicación fue de 35 minutos por prontuario.

**Conclusiones:**

El instrumento construido se demostró viable para basar la auditoría clínica en la identificación del nivel de excelencia o oportunidades de mejoría en procesos asistenciales.

## INTRODUCTION

Hospital accreditation is a voluntary, constant, and restricted standardization process of evaluating the resources of health institutions to guarantee the excellence of care by means of previously accepted standards^(1)^. During this process, the patient’s medical record is one of the documents that must be examined, the importance of its analysis and audit emerging from the fact it clearly shows the care processes related to patient safety, which are effective in the quality management process^(2)^.

The patient record is an essential clinical document in the patient’s diagnostic and therapeutic pathway. It facilitates the provision and continuity of safe care and promotes structured and effective communication between members of the multidisciplinary team, ensuring the quality of care and supporting process improvement, as well as serving as a means of research^(3-4)^.

Thus, medical records are instruments that should be audited to collect and correct waste, irregularities, negligence, and omissions, reflecting the quality of care provided^(5)^. Moreover, their proper management with accurate and comprehensive record keeping is becoming increasingly crucial for hospital administration and judicial demands, as well as in the evaluation of the institution’s level of excellence^(6-7)^.

In this context, clinical audits are seen as a continuous improvement program, which involves collecting information from patient records and bedside. It may be prospective and/or retrospective, going through several cyclical steps^(5)^. As a tool of Clinical Governance, it has the potential to promote change and quality improvements within healthcare organizations, due to its robust method that covers the evaluation of multidisciplinary clinical practice^(8-9)^.

According to the NHS (National Healthcare Service) definition, Clinical Governance is a model for continuously improving the quality of services and ensuring excellence in patient care. The seven pillars of Clinical Governance are: Education; Clinical Audit; Clinical Effectiveness; Risk management; Research and development; Patient experience; Transparency^(10-11)^.

Thus, the clinical audit is a pillar of Clinical Governance that aims to assess and improve clinical performance in relation to previously established standards, resulting in the refinement of clinical practice^(11)^.

The clinical audit of medical records makes it possible to identify problems, define evaluation criteria, collect and analyze data, implement changes, and, finally, to redo the audit of the critical process, among other care processes^(11)^.

Additionally, clinical audits contribute to clinical effectiveness, another pillar of Clinical Governance. Given the current scenario, the focus of health institutions has been to reach a balance between clinical and economic values, that is, to reduce costs and, at the same time, improve care quality and safety^(8)^.

Therefore, clinical effectiveness is defined as the application of the best science-based knowledge obtained, in addition to the clinical experience of professionals, and patient preferences, aiming to achieve excellence in processes and results^(10)^. For this, it is necessary to apply the practice of clinical audits to verify the implementation of measures and standards that the institution has established^(10)^.

By verifying if the assistance provided follows the proper standards and whether the work processes are in conformity with guidelines, the clinical audit allows the development of continuous education with the staff, to guarantee cost reductions regarding the provision of excellent care, bringing no harm to the patient^(9)^.

As an integral part of the quality management system, which is a coordinated activity aimed at managing processes to achieve excellence in meeting patient demands, clinical audits prioritize the criteria of high volume, high risk, high cost, and when relevant, patient involvement in the evaluation of the care provided, focusing on the development of a cultural environment to improve clinical effectiveness, optimizing health outcomes^(11)^. It is noteworthy that audit and feedback are recognized as strategic in improving performance and supporting patient safety in healthcare organizations^(7)^.

One of the priorities of the clinical audit is the Intensive Care Units(ICU), highlighted as units with the highest complexity of structure and care, since they deal with critically ill patients demanding specialized and continuous care; therefore, they have a higher risk of adverse events^(12-13)^. Such events occur due to technical, human, and institutional factors and must be prioritized in the analysis, since critically ill patients have particularities that make them more susceptible to fatal events^(12-13)^.

Given this, it is essential to perform clinical audits in intensive care units to evaluate the clinical practice of the multidisciplinary team and offer managers and health professionals an analysis of clinical performance, efficiency, and clinical effectiveness of the processes standardized by the healthcare organization^(6)^.

The lack of measuring tools to help Clinical Audits is its most relevant shortcoming^(14)^. Developing useful measurement tools to verify the adequacy of knowledge and care processes is a significant step toward ensuring care excellence in health institutions^(15)^. To this end, it is essential to construct and validate the content of the instrument, so that each assessment item is relevant, comprehensive, and representative of what is to be assessed^(15)^.

Thus, the development and content validation of a tool for conducting clinical audits is essential, facilitating the process of analyzing medical records in healthcare organizations. With it, the quality of processes is improved, and it can also help creating action plans to prevent repeated failures, promoting a culture of patient safety and strengthening the continuous improvement programs of the institution.

## OBJECTIVE

To develop and validate the content of an instrument for conducting clinical audits of medical records; to carry out its pre-test.

## METHODS

### Ethical aspects

This study was approved by the Research Ethics Committee of the University where it was carried out.

### Design, period, and place of study

Methodological study carried out in three phases, from May 2020 to May 2021:

1. Development of the instrument from May to September 2020;
2. Content validation with the participation of specialists, from January to March 2021;
3. Application to a sample of 200 medical records, from April to May 2021.

### Study protocol

#### Development of the instrument

To develop the instrument, we considered the criteria proposed by Pasquali: clarity, simplicity, and relevance^(16-17)^. The dimensionality of the construct was also taken into account to evaluate the internal structure and semantics of the instrument and define whether it presents a single component, or different and independent study components^(18)^.

Based on what was described above, the instrument was developed through a bibliographical survey and benchmarking. Benchmarking is a technique that seeks the best practices in the market: it consists in learning from other organizations, seeking more efficient processes and innovative ideas for the institutions to obtain a better performance of their activities^(19)^.

The benchmarking for the development of the instrument was carried out before the beginning of the study using the experience of its leading researcher, who has five years of experience in Quality Management. This is an area responsible for controlling processes and activities within healthcare institutions. Its main goal is to implement good care practices to improve the results of clinical practice^(8)^ in organizations in the state of São Paulo that have active record review committees and hospital accreditation stamps, conferring high reliability and excellence in the care provided. Moreover, they have essential information for clinical audits.

To build the instrument, we proposed domains and assessment items related to care process excellence and patient safety goals in critically ill patients. These choices were based on theoretical references related to clinical governance, patient safety and intensive care^(7-10,12-13)^.

The instrument recommends using a retrospective analysis to evaluate the quality of care provided. Thus, it can be applied to all health professionals who are part of the medical record review committee and/or the patient safety center of the institution.

### Content validation

For content validation, the Delphi technique was used. We invited professionals to be expert evaluators and assessed their agreement in regard to the composition of the clinical audit instrument for medical records.

The Delphi technique is a systematized method with a broad and enriching approach to capturing ideas and knowledge, helpful in obtaining consensus from specialists on a specific subject through validations articulated in phases^(20)^. It is an accessible method because it allows the participation of specialists even if they are physically distant^(20)^. This technique uses consecutive stages to obtain agreement on the content of the instrument. To do so, an e-mail was sent to evaluators, presenting the study and requesting their assessment of the content of the instrument, regarding the clarity and relevance in the wording of each item^(20)^.

As inclusion criteria in the Delphi technique panel, we considered: having experience in management processes and patient safety, being a member or having been a member of the medical record review committee, and being a researcher with experience in the validation of measuring instruments. Professional experience below six months was the only exclusion criterion.

Specialists were identified at the convenience of the researchers, considering those in a business social network, who had participated in quality assessment and patient safety regulating bodies that met the inclusion criteria for the study. The specialists were contacted through instant messaging application groups and business social media.

The group of experts was composed according to content validation references and the Delphi technique^(20-21)^. Seven evaluators who met the inclusion criteria were identified to participate in the assessment of the instrument. The number of specialists varied during the two stages, and two were only present in the first or second stages, leading to a total of nine specialists. The professional category of the group of specialists included higher education professionals (administration and nursing) in the same fields of work: patient safety, management activities, participation in a medical records committee, and quality management in healthcare.

After identification and initial contact via application and social network, the specialists received an e-mail explaining the study’s objectives and the importance of participating according to their experience and professional performance. In the e-mail, they were instructed to judge the relevance and pertinence of the instrument and the concepts involved. After they agreed to participate, the questionnaires were sent through Google Forms and analyzed anonymously^(21)^.

The Delphi technique was performed in two stages to achieve a consensus between the specialists. After we received the instrument from the first stage, a preliminary analysis was performed to determine its composition and the validity of its items. Based on this analysis, a second version was elaborated, incorporating the modifications suggested by the specialists in the first stage. A new e-mail was sent to all those who participated then, including the new version, a summary of the previous stage, and instructions for judging the content to avoid deviating from the main points of the instrument.

### Data collection procedure

The data collection procedure took place in a small private institution in the interior of the state of São Paulo, with demands for clinical and surgical patients, in the Cardiology Intensive Care Unit.

This unit has 12 inpatient beds, and its priority care is for patients in the postoperative period of interventional procedures performed in the hemodynamics unit. The main specialties attended at the institution are cardiovascular, neurology, arrhythmology, and orthopedics (the latter, performed in the Surgical Center). In addition, the institution is a reference for clinical patients from other institutions in the region.

The pre-test was performed based on 200 medical records from 2020 and 2021. The choice of these records followed the eligibility criteria: patients with a length of stay > 3 days; patients who have undergone different lines of care (Emergency; Intensive Care Unit; Surgical Center; Inpatient Units); patients ≥ 60 years with multiple comorbidities; and patients elected for guideline-based management (sepsis, chest pain, stroke, and/or palliative care). Due to the pandemic period in which the pre-test of the instrument was conducted, the medical records made available by the Medical Records Committee of the institution were analyzed by convenience. The sample size calculation followed the recommendation of a statistical professional, who oriented to use between five and ten evaluations per item.

A term of responsibility was sent to the institution for appreciation and authorization as to the pre-testing of the instrument, which was built to consult the medical records. The instrument was applied by the lead researcher of the study, a member of the medical records review committee of the institution under study. The database is available through the following link: https://doi.org/10.25824/redu/PTAPUA

### Analysis of the results and statistics

Besides evaluating the agreement on the domains and items of the instrument, the Content Validity Index (CVI) was evaluated, in which the specialists attributed to each item one of the four options:

1. Not relevant or not clear for medical record audits;
2. Needs major revisions to be relevant or clear for record audits;
3. Needs minor revisions to be relevant or clear for record audits;
4. Relevant and representative for record audits.

To calculate the CVI for each item, the total number of “3” or “4” options assigned by the specialists was considered, divided by the total number of answers; items with a CVI higher than 0.90 were considered valid^(21)^.

For the pre-test analysis we used descriptive statistics, which is a means of organizing and summarizing the main characteristics observed in a set of data, allowing the researcher a better understanding of the data studied^(22)^.

## RESULTS

The initial development of the instrument was composed of 11 domains, each with sub-items that characterized the quality of care provided by the multidisciplinary team.

To reach a content validity index above 0.90 in all sub-items of the domains, two stages of the Delphi technique were required, as shown in Table 1. In the first stage, the judges gave suggestions for more clarity in the domains “Device-Related Infections,” “Managed Care Risks,” “Multidisciplinary Team Actions,” and “Safety Culture.”

**Table 1:** Content Validity Index, in the two phases of the Delphi technique (n = 7), Campinas, São Paulo, Brazil, 2021

Domain	Phase I Delphi	Phase II Delphi
Patient Identification	1.0	
Actual Communication	1.0	
Safety in Medication Prescription, Use, and Administration	1.0	
Safe Surgery	1.0	
Device-Related Infections	0.86	1.0
Managed Care Risks	0.86	1.0
Guideline-Based Protocols	1.0	
Medical Procedures	1.0	
Nursing Procedures	1.0	
Multidisciplinary Team Actions	0.86	1.0
Safety Culture	0.86	1.0

Based on the suggestions, a new version of the domains and items was presented in the second phase of the Delphi technique, for content validation.

After content validation and the final version of the “Clinical Audit of Medical Records” instrument, 200 medical records were audited. The assessment time varied between 30 and 40 minutes per record. The pre-test of the instrument is presented, per domain, in Tables 2, 3, and 4.

**Table 2:** Pre-testing of the instrument and analysis of medical records regarding Patient Identification, Effective Communication, Safety in Medication Prescription, Use, and Administration, and Safer Surgery, Campinas, São Paulo, Brazil, 2021

Item/information	1^*^	2+	3ǂ	4§	NAǁ
Patient Identification					
Admission form	0	0	0	100	0
Exhibition of an identification wristband	5.5	42.5	42	10	0
Documents completed with patient identification	0	12.5	52.5	35	0
Signed Informed Consent Form	1.5	1.5	0	97	0
Clear reason for admission	0	0.5	0.5	99	0
Therapeutic plan at admission	13.5	18.5	4.5	63.5	0
Other patients' documents in the medical record	0	0	0	0	100
Actual Communication					
Communication process between sectors (transition of care)	2.5	4.5	15.5	77	0.5
Records of the interdisciplinary visit	6	12	23.5	58.5	0
Safety in Medication Prescription, Use, and Administration					
Medication reconciliation by a pharmacist (first 24 hours)	71	6.5	14	8.5	0
Reconciled prescription drugs	71.5	9	11.5	8	0
Double-checking of high surveillance drugs	35	37	24	4	0
Safe Surgery					
Informed Consent Form for surgical procedures	1.0	3.0	6.0	58.5	31.5
Informed Consent Form for anesthetic procedures	0.5	1.5	4.5	38	55.5
Surgical description	19.5	0.5	1.0	47.5	31.5
Safe Surgery Checklist	0	0	3.0	65.5	31.5
Evidence of surgical antibiotic prophylaxis (up to 60 minutes before the surgical incision)	0.5	0	0	40.0	59.5

Caption:

* Lack of information; + Inaccurate and/or incomplete information; ǂ Clear but incomplete information; § Accurate and complete information; ǁ Not applicable.

**Table 3:** Pre-testing of the instrument, analysis of medical records regarding Device-Related Infection Prevention Measures, Managed Care Risks, Managed Protocols, Campinas, São Paulo, Brazil, 2021

Item/information	1^^*^^	2^+^	3^ǂ^	4^§^	NA^ǁ^
Device-Related Infection Prevention Measures					
Ventilator-associated pneumonia	0	3.5	3.5	2.5	90.5
Central venous catheter bloodstream infection	0.5	11	3.5	1	84
Urinary tract infection associated with the use of indwelling urinary catheter	0	19.5	12	4.5	64
Managed Care Risks					
Braden Scale	0	0.5	2	97.5	0
Morse Scale	0	0.5	0.5	99	0
Identification of risk for bronchoaspiration	2.5	1	0	92.5	4
Prevention measures for thromboembolism	9.5	0	0.5	0	90
Bleeding surveillance in surgical patients	0.5	1	0.5	68.0	30.0
*Delirium during hospitalization*	48.5	9.5	2	23	17
Clinical Deterioration	0	0	0.5	0.5	99
Prescription of preventive measures	0	20	27	53	0
Record of preventive measures	0	2.5	19	78.5	0
Guideline-Based Protocols					
Criteria for stroke identification	0	0	0	10	90
Criteria for identifying chest pain	0.5	0.5	0	18.5	80.5
Eligibility criteria for palliative care	0.5	0	0	3	96.5
Protocols managed at recommended times	0	2	0.5	25.5	72
Guideline-based protocols related to the principal diagnosis	0.5	0	0	3.0	96.5
Guideline-based protocols with same outcomes	0	0	0	31	69
Outcome in accordance with primary diagnoses	0	0.5	0	99.5	0

Caption:

* Lack of information; + Inaccurate and/or incomplete information; ǂ Clear but incomplete information; § Accurate and complete information; ǁ Not applicable.

**Table 4:** Pre-testing of the instrument, analysis of medical records regarding Medical Procedures, Nursing Procedures, Multidisciplinary Team Actions, and Safety Culture, Campinas, São Paulo, Brazil, 2021

Item/Information	1^^*^^	2^+^	3^ǂ^	4^§^	NA^ǁ^
Medical Procedures					
Description of the reason for hospitalization at admission	0	0.5	0	99.5	0
Medical evolution coherent with the therapeutic plan	2	10.5	7	80.5	0
Discharge summary with clear information about what happened with the patient during hospitalization	2.5	2	1	94.5	0
Medical prescriptions carried out every day during hospitalization	0	0	0	100	0
Nursing Procedures					
Care plan carried out by the nursing team	1	0	0	99	0
Clear nursing care plan, related with the reason for hospitalization	0.5	15.5	27	57	0
Nursing process applied every day of hospitalization	0	0.5	0.5	99	0
Multidisciplinary Team Actions					
Prescribed physical therapy is registered in the evolution of the attention	1.5	0	12	86.5	0
Prescribed speech therapy is registered	57	21.5	5	8.5	8
The evolution of occupational therapy care is registered	0	0	0	0.5	99.5
When there is nutritional evaluation, records are made in case of nutritional risks	47	1	0.5	3.5	48
Psychological attention, when requested, is registered	11.5	0.5	1	0.5	86.5
Social services, when requested, are registered	0	0	0	0	100
Safety Culture					
Are patient safety protocols respected?	0	17	46	37	0
All printed copies of the records are signed by the professional responsible for care	0	4	11	85	0
Is there evidence, in the medical records, of complications or adverse events?	0	0	0	4	96

Caption:

* Lack of information; + Inaccurate and/or incomplete information; ǂ Clear but incomplete information; § Accurate and complete information; ǁ Not applicable.

Table 2 presents data from the analysis of medical records related to Patient Identification, Effective Communication, Safety in Medication Prescription, Use, and Administration. Table 3 shows the data extracted regarding Device-Related Infection, Care Risks, Guideline-Based Protocols, and Medical Procedures. Table 4 shows the data extracted using the instrument to analyze medical records related to Medical Procedures, Nursing Procedures, Multidisciplinary Team Actions, and Safety Culture.

## DISCUSSION

The development and content validation of an instrument for clinical auditing was a challenging trajectory in which we sought to contribute to clinical and scientific practice by providing an innovative instrument that could make tangible and measurable the quality/safety registered in medical records. To do so, it was necessary to rely on references about the development and validation of instruments^(1-2,15,21)^, and on the knowledge and experience of professionals involved in the auditing of medical records and quality management in healthcare.

In the process of building the instrument, benchmarking was also a strategy to allow the application of the instrument in different public and private institutions, since benchmarking enables continuous improvement, aiming to enhance the performance of the institutions’ critical processes^(19)^.

Among the criteria necessary to build a good measuring instrument, we emphasized objectivity and clarity, i.e., long sentences with full details can provide a tiring and unfocused reading^(23)^. Therefore, taking into account the clinical audit process to standardize the evaluation of medical records and make it more objective, the specialists’ considerations in the two phases of the Delphi technique enabled reformulations in the domains and items of the validated instrument, in order to analyze the conformity of the information registered in medical records^(20)^.

Content validation is the first stage in the development of measurement tools, and having achieved a CVI higher than 0.9 for all items of the tool indicates an excellent level of content validity regarding the clarity, readability, relevance, and pertinence of each item assessed^(15)^.

The process of content validation also has associations with the scope of the instrument^(16-17,20)^. Thus, the application of the instrument should be prioritized in the clinical audit of the medical records of chronically ill or critically ill patients, due to the composition of the items built and to the potential it presents for the management of processes, and of financial and human resources inherent to the complexity of care for this profile of patient^(24-26)^.

In the pre-test of the instrument, after the content validation was completed, the average application time of 35 minutes per medical record showed that the application of the tool is feasible in the clinical audit process^(7,15-20)^.

The process of validating an instrument should be continuous, so construct evaluation, validity, reliability, homogeneity, and equivalency should be continued in the institutions that adopt it^(15)^. This study had important limitations, such as the impossibility to perform inter-evaluator reliability. This limitation was due to the fact that data collection took place during the social distancing period enforced to contain the spread of SARS-CoV-2, reason why the Ethics Committee did not approve the initial study protocol for simultaneous data collection by two researchers.

Additionally, the application of the instrument in different institutions was not authorized due to the pandemic that took place in the period of data collection. This prevented the performance of exploratory factor and reliability analyses of the internal consistency using Cronbach’s alpha^(27)^.

It is noteworthy that, in the pre-test, it was possible to experience the importance of clinical auditing in the analyses of care processes, enabling the discrimination of elective processes for improvement of consolidated processes, i.e., the tool was perceived to be adequate for the assessment of the maturity of care processes.

Therefore, the application of the instrument validated in this study is recommended to standardize the auditing of medical records, so hospitals can establish a method for continuous improvement and diagnose which are their best-defined, mature, and organized processes, aiming at excellence in care. Thus, its application may bring many benefits, from the perception of quality and safety by the multidisciplinary team to the process of hospital accreditation, which presupposes the implementation of good care practices based on quality management and structural changes^(28-30)^.

The aggregation of theoretical references, knowledge of professional specialists, benchmarking in the development, and validation of the content of the clinical audit instrument built in this study allowed us to make available a tool for hospital institutions to standardize the evaluation of medical records and thus analyze interdisciplinary processes and practices that ensure the quality of care in a manner committed to continuous improvement.

### Study limitations

As a limitation of this study, it should be noted that due to the pandemic during the period of data collection, the inter-evaluator reliability analysis of the clinical audit instrument was not authorized, despite being recommended before its implementation.

### Contributions for the field of nursing, health, or public policy

The development and content validation of an instrument for clinical audits will optimize the management of the work process regarding patient safety and quality of care for critically ill patients, since these clients require complex care and are exposed to many significant care risks. Therefore, having a tool that guides the active search for processes that need to be improved is of great importance in the work process of quality management and for patient safety, which together have a single goal: to develop good practices to provide excellent care, free of harm.

The clinical audit, through the instrument validated in this study, makes it possible to base training and action plans on the processes that need to be improved and, thus, provides a program for continuous improvement within health institutions, aiming at the reduction of avoidable adverse events, at focused care, and at a good patient experience.

## CONCLUSION

The study allowed us to build and validate the content of an instrument for conducting clinical audits of medical records. All items presented a CVI higher than 0.9, which denotes an adequate content validation.

The participation of specialists involved in quality management in health care in the process of content validation enhanced the composition of the clinical audit instrument in 11 domains and 52 evaluation items.

The pre-test of the clinical audit instrument in 200 medical records with an average application time of 35 minutes per record shows the feasibility of this tool in daily work.

Therefore, the use of the instrument is recommended to evaluate the maturity of care processes and to establish continuous improvement programs in healthcare institutions.
